# Novel insight into miRNA biology and its role in the pathogenesis of systemic lupus erythematosus

**DOI:** 10.3389/fimmu.2022.1059887

**Published:** 2022-12-02

**Authors:** Baiwei Luo, Kaixia Zhou, Yingcong Liufu, Xia Huang, Huiqiong Zeng, Zhaoyang Zhang

**Affiliations:** ^1^ Department of Rheumatology and Immunology, Yuebei People’s Hospital Affifiliated to Shantou University Medical College, Shaoguan, Guangdong, China; ^2^ Department of Rheumatology, Shenzhen Futian Hospital for Rheumatic Diseases, Shenzhen, Guangdong, China; ^3^ Department of Clinical Laboratory, Shanghai Cancer Center, Fudan University, Shanghai, China; ^4^ Shanghai Institute of Rheumatology, Renji Hospital, Shanghai Jiao Tong University School of Medicine, Shanghai, China; ^5^ Department of Anorectal, Shenzhen TCM Anorectal Hospital (Futian), Shenzhen, China; ^6^ Department of Xi Yuan Community Health Service Center, The Eighth Affifiliated Hospital of Sun Yat-sen University, Shenzhen, Guangdong, China

**Keywords:** miRNAs, unconventional function, immunity, epigenetics, systemic lupus erythematosus

## Abstract

MicroRNAs(miRNAs) have emerged as key regulators that control and influence gene expression as well as multiple biological processes depending on their potential binding sites in human-protein coding genes and other unconventional patterns, including coding for peptides, activating Toll-like receptors as a ligand, and other manners. Accumulating evidence has demonstrated that microRNA expression is tightly regulated during phases of development, differentiation, and effector functions of immune cells, immunological disorders of systemic lupus erythematosus (SLE). This review outlines the biogenesis of miRNAs and their unconventional functions as well as underlying cellular and molecular mechanisms. It then summarizes our current knowledge about how the biogenesis of miRNAs is regulated. Moreover, an overview was provided concerning the role of abnormal expression of miRNAs in lupus immune cells. In particular, we will shed some light on the recent advances in the role of miRNAs and exosome-derived miRNAs in immunological and epigenetic pathways in the pathogenesis of SLE.

## miRNA biogenesis

MicroRNAs (miRNAs) are a novel class of endogenous, single-stranded RNAs of approximately 19–25 nucleotides in length, which are synthesized from an endogenous hairpin- shaped transcript by RNase-III-type enzyme (1). In 1993, Victor Ambros and colleagues investigated defects in the temporal control of Caenorhabditis elegant and discovered the first miRNA called lin-4 (2). During the last decades, miRNAs have been listed in large numbers in eukaryotes of plants and vertebrates, and since then, investigation of the biogenesis and processing of miRNAs has brought numerous insights into physiopathology.

**Figure 1:**
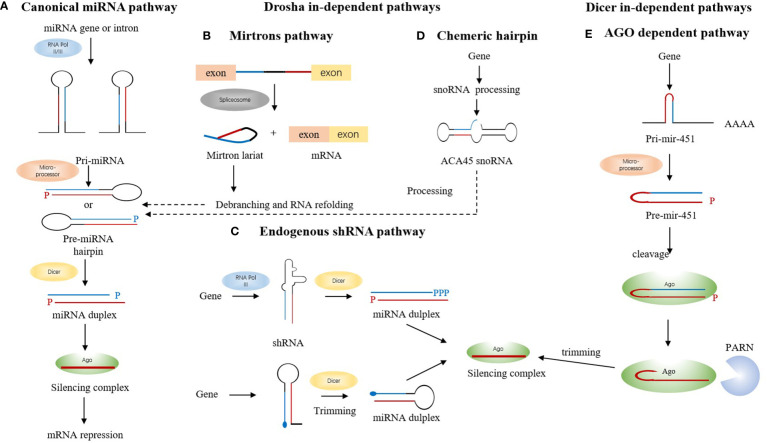
The biogenesis of miRNA. **(A)** miRNA genes are transcribed to pri-miRNA, which are then processed by a microprocessor complex to generate pre-miRNA. After getting exported to the cytoplasm *via* exportin-5, pre-miRNAs are subsequently processed to mature miRNAs by RNase III Dicer and then loaded onto the Ago to form the effector RISC, which represses translation. **(B)** Mirtrons are located in the introns of the mRNA encoding host genes, which arise from the spliced-out introns. The short hairpin introns use splicing to bypass Drosha cleavage. Mirtron lariat can enter the canonical miRNA pathway after debranching and RNA refolding. **(C)** After getting trimmed by Dicer, shRNA forms the miRNA duplex and then gets transported onto Ago to form the silencing complex. **(D)** snoRNAs like ACA45 snoRNA is processed into canonical miRNA pathway ultimately associated with Ago 1 and 2. **(E)** miR-451 is processed by microprocessor complex and does not require Dicer.

Studies have illustrated that miRNA is a multifunctional small molecule and its biogenesis through a series of pathways and sequential processes starting with the cleavage of the primary miRNA transcript in the nucleus by the Microprocessor complex (3). Canonically, miRNA genes are transcribed to generate long primary transcripts (pri-miRNAs), which are then processed by RNase-III-type enzyme Drosha to release the hairpin-shaped pre-miRNAs within the nucleus. Drosha functions together with its essential co-factor DGCR8/Pasha known as the Microprocessor complex (4). Next, Pre-miRNA gets exported to the cytoplasm *via* exportin-5, a member of the Ran-dependent karyopherin family (5). After arriving in the cytoplasm, pre-miRNAs are subsequently processed to mature miRNAs by RNase III Dicer and loaded onto the Argonaute (Ago) protein to form the effector RNA-induced silencing complex (RISC), which can be programmed to target virtually any nucleic acid sequence for silencing (1) (**Figure 1A**).

Drosha and Dicer are classes I and Ill members, respectively, of the RNase Ill protein family that play a key role in miRNA biogenesis (6, 7).Functionally, both proteins act as a “ruler” and “scissors” as they measure and cut at a fixed distance of each substrate (8, 9). The overall structural and enzymatic reaction similarity of both proteins, in the substrate and its binding mechanisms, suggests that both proteins share dynamics similarity related to their common functions. In the nucleus, Drosha together with its cofactor DiGeorge Syndrome Critical Region 8 (DGCR8) constitute a complex known as Microprocessor. The complex excises a long primary transcript (pri-miRNA) to release hairpin shaped precursor-miRNA (pre-miRNA) of ~70 nucleotides in length with a 3’overhang. The pre-miRNA is subsequently cleaved at the cytoplasm by Dicer, yielding a miRNA duplex of ~22nt in length, when one strand of this duplex remains as a mature miRNA, while the other strand is degraded (10, 11).

In recent years, many studies shed some light on other novel mechanisms to produce functional miRNAs apart from the canonical one (12, 13). Some certain debranched small introns can mimic the structural hallmarks of pre-miRNAs to enter the miRNA-processing pathway, named “mirtrons”, thus bypassing the need for assembling into a Microprocessor complex (14). Mirtron hairpins are processed by the action of the cellular splicing machinery and lariat-debranching enzyme before merging into the canonical miRNA pathway during nuclear export by Exportin-5, and are subsequently processed by Dicer-1 (15). (**Figure 1B**) Startlingly, some studies showed that endogenous short hairpin RNAs were processed into miRNAs (mir-320 and mir-484). Further examination of the sequence and structure of these short hairpin RNA (shRNA)-derived miRNAs showed the lack of microprocessor-binding sequence, which further demonstrates its Dgcr8 independence (13)(**Figure 1C**). The third one comes to an exploration of some of the small nucleolar RNAs (snoRNAs), which were reported to interfere with the gene expression process at the levels of mRNA stabilization and translation by binding with Ago (16). Deep sequencing data of selected snoRNAs associated with Ago 1 and 2 identified snoRNAs as another source of miRNA that may follow the canonical pathway in some cases (17) (**Figure 1D**). The examination of miR-451and its potential targets directs the conclusion that this miRNA is processed by Drosha yet its maturation does not require for Dicer. Instead, the pre-miRNA was loaded into Ago and cleaved by the Ago catalytic centre to generate an intermediate 3′ end, which is then further trimmed to mature miRNA by the 3′–5′ exonuclease poly(A)-specific ribonuclease PARN (18) (**Figure 1E**).

## Unconventional function of miRNA

Most genetic and biochemical studies to date have shown that miRNAs induce translational repression by binding to the 3′ untranslated region (3’ UTR) in their target mRNA (3). However, there are accumulating evidences showing that miRNAs can function outside this paradigm. The miRNAs, usually, are first transcribed as primary transcripts with a cap and a poly-A tail and processed to stem-loop precursor miRNAs (19). Conventionally, the 5’ cap helps mRNA prevent degradation by hydrolytic enzyme and function as part of “attach here” sign for ribosomes in the cytoplasm. The poly-A-tail inhibits the degradation of RNA and helps ribosomes attach and facilitates the export of mRNA from the nucleus. Recently, bioinformaticians used ORF finding tools to predict the protein-coding potential of non-coding RNAs, and discovered unexpectedly that many more pri-miRNAs contain short open reading frames (ORFs). Further, Lauressergues et al. initially discovered that pri-miR-171b of Medicago truncatula and pri-miR-165a of Arabidopsis thaliana can produce short peptides, named as miPEP-171b and miPEP-165a separately. Functional studies proved that these miPEPS increase transcription of their own pri-miRNA, which ultimately raise the accumulation of their corresponding mature miRNAs (20). Soon after, Fang et al. demonstrated that, in human, several pri-miRNAs, including pri-miR-200a and pri-miR-200b can produce short peptides which regulate the migration of cancer cells (21).

Toll-Like Receptors (TLRs) play a critical role in the early innate immune response to invading pathogens by sensing microorganism and are also involved in sensing endogenous danger signals. Previously, mmu-miR-21 and hsa-miR-29a were reported to activate TLR8 (in human, and TLR7 in mice) as ligands in immune cells, triggering a TLR-mediated prometastatic inflammatory response that may ultimately lead to tumor growth and metastasis (22). Almost at the same time, Lehmann et al. uncovered that extracellular let-7, a miRNA derived from the central nervous system, activates the RNA-sensing TLR 7 and led to neuronal apoptosis through neuronal TLR7. Moreover, cachexia (microvascular miR-21) and sepsis (Kaposi Sarcoma-associated Herpesvirus miRNAs) can block these effects (23, 24). miRNAs commonly regulate mRNA repression by associating with ago protein. It was reported that, however, in blast crisis chronic myelogenous leukemia (CML-BC), apart from targeting mRNA encoding the survival factor PIM1, restoration of miR-328 can also interact with the translational regulator poly (rC) -binding protein hnRNP E2 and then leads to release of CEBPA mRNA from hnRNP E2-mediated translational inhibition. It’s interesting that the miR-328 interaction with the functional proteins hnRNP E2 is not depending on the miRNA’s seed sequence (25).

Argonaute2 protein (AGO2) plays a key role in a variety of pathophysiological processes by participating in the formation of RISC with ribonucleic acid endonuclease activity and by promoting the maturation of microRNAs and regulating their biosynthesis and function, thereby inhibiting the expression of target genes (26, 27).

Moreover, in stressed hepatic cells, HuR was found to reversibly bind miR‐122 to substitute them from Ago2 and subsequently gets freed to ultimately influence the stress response in starved human hepatic cells (28). Most investigation into metazoan miRNA function has been for sites in 3′ UTRs, experiments using artificial sites, however, showing that targeting can also occur in 5′ UTRs and open reading frames (ORFs). Furthermore, it’s interesting to find that miR-10a can enhance mRNAs translation by interacting with the 5’ UTRs of their encoding ribosomal protein (29, 30). Lastly, researchers systematically surveyed the secondary structure of 5′ UTRs in both plant and vertebrates and found a universal trend of increased mRNA stability that are regulated by miRNA targeting the 5′ cap in mRNAs (31)

In addition, Hwang et al. (2007) demonstrated that miR-29b has a function of directing nuclear enrichment of small ncRNAs to which it is attached by a distinctive hexanucleotide terminal motif that acts as a transferable nuclear localization element (35). miR-589 was found to bind the promoter RNA and then activated COX-2 transcription (33) Interestingly, several studies have reported that nuclear miRNAs can enter and target the mitochondrial genome, for instance, miR-181c translocates into the mitochondria, remodeling electron transport chain complex IV and causing mitochondrial dysfunction (36). Other than these, Tang et al. provides evidence that one miRNA can directly target their primary transcripts to control the biogenesis of other miRNAs in the nucleus (37).

MiRNA plays a key role in the pathogenesis of SLE by regulating the expression of post-transcriptional genes through a variety of unconventional pathways. **Table 1** lists miRNAs that are preliminarily reported as unconventional miRNA functions. Specific miRNA dysregulation is closely related to the occurrence and development of SLE. Therefore, detecting or regulating the abnormal expression of miRNA in SLE and other immune-related diseases is very important for the clinical diagnosis, prediction and treatment of SLE.

**Table 1:** Unconventional miRNA functions.

Pri-miRNAs Coding for Peptides			
miRNA	Peptide	Implicated function	ref
mtr-pri-miR171b ath-pri-miR-165a	miPEP171b miPEP165a	increasing transcription of their own pri-miRNA, which subsequently enhances the accumulation of their corresponding mature miRNAs	(32)
Hsa-pri-miR-200a Hsa-pri-miR-200b	miPEP-200a miPEP-200b	regulate migration of cancer cells	(21)
**miRNAs activating Toll-like receptors**			
miRNA	Target	Implicated function	ref
mmu-miR-21 hsa-miR-29a	TLR8 (murine) TLR7	triggering a TLR-mediated pro-metastatic inflammatory response that ultimately may lead to tumor growth and metastasis	(22)
Hsas-let-7b-5p	TLR7	activates the RNA-sensing Toll-like receptor (TLR) 7 and induces neurodegeneration through neuronal TLR7	(24)
**miRNAs Interacting with Non-AGO Proteins**			
miRNA	Interacting partner	Implicated function	ref
Hsa- miR-328	hnRNP E2	when miR-328 is re-introduced it can rescue differentiation and impair survival of leukemic blasts in chronic myelogenous leukemia	(25)
Hsa-mir-122	HuR	HuR could reversibly bind miR-122 to replace them from Ago2, which controls stress response in starved human hepatic cells	(28)
miR-589	Promoter RNA	Binds the promoter RNA and activates COX-2 transcription.	(33)
**miRNAs targeting outside the 3′UTRs**			
miRNA	Target site	Implicated function	ref
miR-10a	5′ UTRs	interacts with the 5’ untranslated region of mRNAs encoding ribosomal proteins to enhance their translation	(29)
miR-29b	miRNA or siRNA	The distinctive hexanucleotide terminal motif of miR-29b acts as a transferable nuclear localization element that directs nuclear enrichment of miRNAs or small interfering RNAs to which it is attached.	(34)

## Modulation of microRNAs processing and expression

miRNA biogenesis has to be tightly regulated to guarantee a suitable number of functional miRNAs since they are crucial for cellular development and homeostasis. The mechanisms of regulating miRNA biogenesis are similar to other RNAs, such as transcriptional activation or inhibition, processing and regulating maturation, miRISC and target regulation, RNA editing, and so on.

Transcription is a major point of regulation in miRNA biogenesis and the transcription of miRNAs is under the control of numerous transcription factors. For instance, Pol II-associated transcription factors, like myogenin and myoblast determination 1(MYOD1), bind upstream of miR-1 and miR-133 loci and trigger the transcription of these miRNAs during myogenesis (38). Some miRNAs are under the control of tumor-suppressive or oncogenic transcription factors. The miRNA family of miR-34 can be activated by the tumor suppressor p53 (39), while the amount of miRNA related to apoptosis and cell cycle can be trans-activated or repressed by oncogenic protein MYC (40).

Post-transcriptional regulation is another major point of regulation in miRNA biogenesis, which affects Drosha and Dicer processing, as well as miRNA modification and turnover. Drosha and Dicer and RISC loading is the key step for miRNA precursors processing, and this processing can be facilitated, supported, or inhibited by tons of factors (41). ADAR1 and ADAR2 can edit specific adenosine residues of some certain miRNA precursors. For example, editing of the precursor of miRNA-142, induce the suppression of its processing by Drosha. When the pri-miR-142 is edited, it can be degraded by Tudor-SN, a component of RISC and also a ribonuclease specific to inosine-containing dsRNAs (42). Increasing evidence suggests that general RBPs, including splicing factors and other diverse RNA processing factors, act as post-transcriptional regulators of miRNA processing.

Recent studies have reported that not only the coordination of individual miRNA processing steps, but the connection of miRNA biogenesis with other cellular processes is involved with regulatory factors (43). For instance, protein phosphorylation links miRNA biogenesis to various signaling pathways, and has countless positive connections to diseases. The results mentioned above heighten that even presumably well-understood processes might be far more complex and that further work is needed to explore miRNA biogenesis (41). Innovatively, Hong Chang et al. expanded their observation to the 3′ region of pri-miRNA, and found that miRNA biogenesis process can be unbiasedly impeded by targeting both 3′ and 5′ regions (44).

Epigenetic control also contributes to miRNA gene regulation; miR-203 undergoes DNA methylation in the T-cell lymphoma, while avoiding modification in normal T lymphocytes (45). DNA methylation is the major modification method in eukaryotic genomes, which can downregulate gene expression. In different developmental stages or under different pathological conditions, the overall methylation degree of CpGs can fluctuate in mammals (46). Some researchers have reported that aberrant methylation of CpG islands adjacent to their promoters can silence some tumor suppressor miRNAs, such as miR-203 (47, 48). A recently new discovered modification of CpG dinucleotides is DNA hydroxymethylation, which associates with the addition of a hydroxyl group on 5-methylcytosines (5mCs) to produce 5-hydroxymethylcytosine (5hmCs) (49, 50).

Bifunctional RNAs could interact with miRNAs (51). After the statistical analysis of miRNA fold change of expression in response to Drosha knockdown and miRNA secondary structure, Henrik Sperber et al. make a conclusion that the absence of mismatches in the central region of the hairpin, 5 and 9-12 nt from the Drosha cutting site conferred decreased sensitivity to Drosha knockdown. Changes in Drosha expression can be a biologically relevant mechanism, through which process, eukaryotic cells could control miRNA profiles (52).

The expression process of miRNA is regulated by many factors, and the change of any link will lead to the abnormal expression of miRNA, and then result in the corresponding diseases. Therefore, a detailed understanding of the regulation of miRNA expression process is of great significance for understanding the occurrence mechanism of SLE and other related diseases and finding corresponding solutions.

## miRNAs in the pathogenesis of SLE

SLE is the prototypic as well as chronic autoimmune disease, which is characterized by multiple autoantibodies associated with a multisystem illness. A broad spectrum of clinical manifestations including skin rash, photosensitivity, oral ulcers, arthritis, serositis, glomerulonephritis, neurological symptoms (e.g. seizures), leukopenia, and thrombocytopenia. During the last decade, a multidisciplinary approach applied in SLE-relevant research has built a more concise view of this disease. Additionally, collective evidence supports the view that various SLE-relevant processes can be affected by miRNAs.

### Pathogenesis of systemic lupus erythematosus

In SLE, patients exist some characteristics like a lack of tolerance against nuclear autoantigens, polyclonal autoantibody production, immune complex deposition, and tissue inflammation. The pathogenesis of SLE is extremely complex; genetic, epigenetic, environmental, and immune-regulatory factors can jointly contribute to disease development. Although SLE has been investigated extensively and deeply for decades, however, the exact etiology of SLE remains unclear. In past decades, using genetic variant identification, expression pattern analysis, and mouse models, as well as epigenetic analysis, researchers have made further understanding of SLE. Taken together, most of these findings direct to versatile functions of endogenous miRNAs in innate immune responses, immune and resident cell dysfunction, and the association between abnormal epigenetic regulation and SLE, which have attracted considerable interest worldwide separately and together.

### microRNAs in the modulation of immunity of SLE

The immune system is made up of an elaborate network of cells, tissues, and organs that work together to protect the body from invaders (bacteria, viruses, fungal infections, and parasites). Due to their highly evolutionary conserved nature and their wide range of regulating effects, miRNAs are emerging as a critical part of the development and function of the immune system, both innate and adaptive compartments. Various evidence had proved that derailments of both the innate and adaptive immune systems contribute to the pathogenesis of SLE, and it is not surprising that almost all SLE-relevant processes can be affected by miRNAs (53). During the immune pathogenesis of SLE, immune cells build a complex signal network as a core component, and miRNA influences disease progression by regulating immune cell function. In the next part, we focus on miRNA-mediated immune cell dysfunction associated with SLE.

Macrophage polarization was found to affect the initiation and perpetuation of SLE. During activated lymphocyte-derived DNA (ALD-DNA) induced macrophage M2b polarization, dynamic miRNA expression patterns and network analysis are described. Around 11% miRNAs were differentially expressed in macrophage M2b polarization. Differentially regulated miRNAs at 6 h are significantly associated with inflammatory response and disease, while miRNAs at 36 h had an effect on cell proliferation (54–56). Differentially expression of miRNA directs to the macrophage polarization which then contributes to the pathogenesis and progression of SLE and may provide potential targets for therapies.

The interferon signature is present in the blood of patients with SLE, especially younger patients and those of Asian ancestry (57). Type I IFN(IFN-I) overexpression is considered as a significant feature of SLE. As a central regulator of immune responses, DCs is also confirmed to play a pivotal role in the pathogenesis of SLE, mainly through IFN-I production. miR-146a and miR-155 and their star form partner miR-155* cooperatively regulate the IFN-I production in human pDCs (58). miR-146a is a negative regulator of IFN-I pathway by targeting interferon regulatory factor 5 (IRF5), interleukin-1 receptor-associated kinase (IRAK) 1and tumor necrosis factors receptor associated factor (TRAF) 6 in SLE (59).

Lately, it was found that miR-142-3p is involved in regulating the proinflammatory function of monocyte-derived DCs in the process of SLE. miR-142-3p was verified among the highly expressed miRNAs in the SLE group and overexpressing miR-142-3p in moDCs of the NC group caused an increase of SLE-related cytokines, such as CCL2, CCL5, CXCL8, IL-6, and TNF-α. But the underlying mechanism of how this function is associate with CCL2, CCL5, CXCL8, IL-6, and TNF-α still remains unclear (60).

Study shown that miR-124 is downregulated in patients with active lupus nephritis(LN), resulting in increased expression of its mRNA target, TRAF6 (61). miR-130b-3p affects classically activated macrophage (M1) macrophage polarization by suppressing IRF1 (62). Hsa-miR-127-3p, a microRNA whose expression is downregulated in renal tissues of LN patients, is associated with upregulation of Janus kinase 1 (Jak1) and IFN stimulated genes (ISGS) in renal tissues of LN patients, leading to hyperactivation of the IFN-I signaling pathway in the kidneys of LN patients (63).

**Figure 2:**
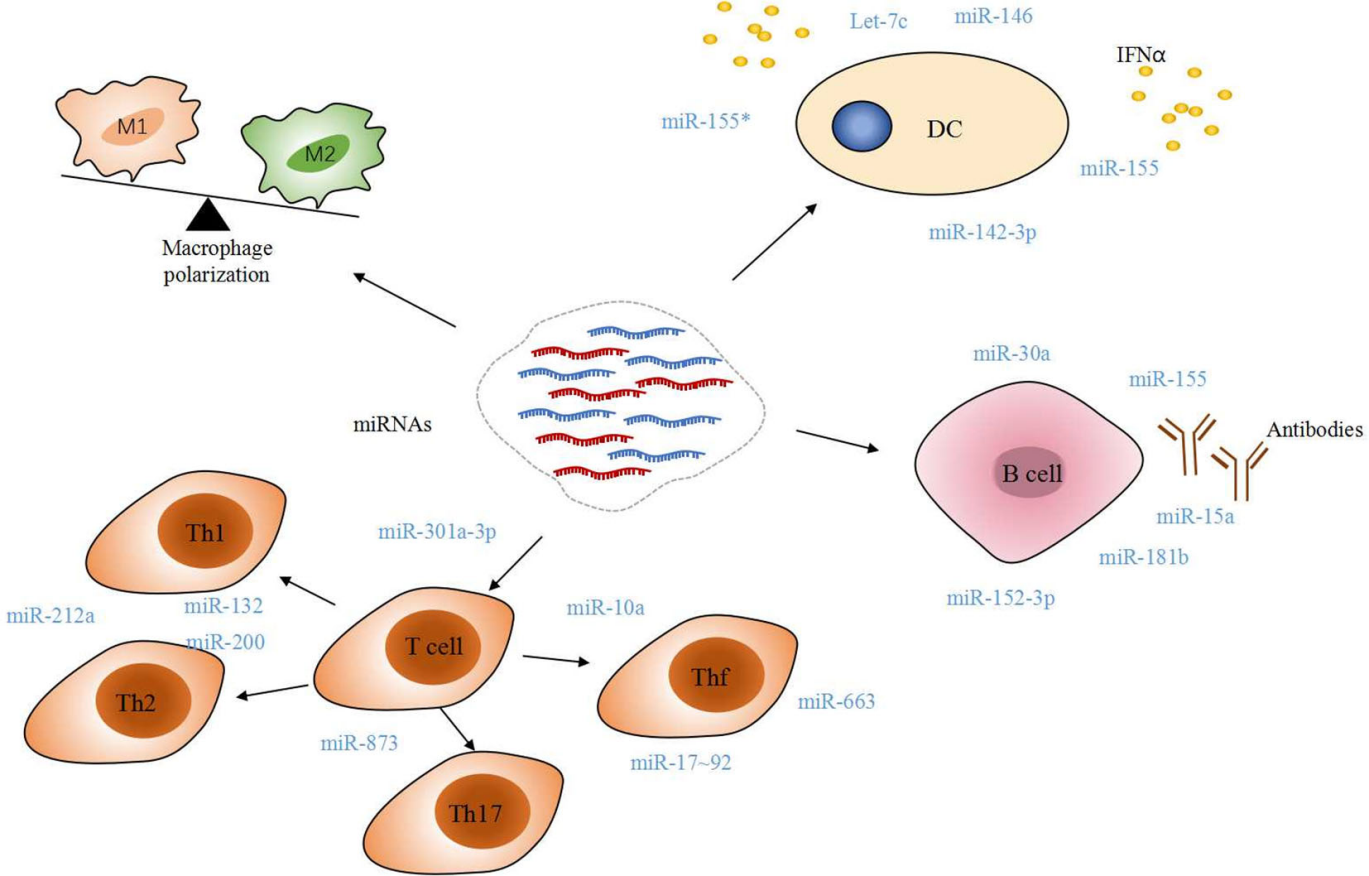
Immune modulatory miRNAs involved in SLE. Dysregulated macrophage polarization and T cell differentiation partially blame on miRNAs. A mass of miRNAs would trigger IFNα released in DC, B-cell self-reactivity and autoantibody production. All of these manners contribute to the pathogenesis of SLE, directly or indirectly.

Over the last few years, with the dominant role of T cells in autoimmunity being established, the concept that abnormal differentiation and impaired function of T cells contribute to various SLE-relevant processes was gradually accepted by people. It is clear that cytokines and chemokines present in the inflamed microenvironment regulate the differentiation and function of various subsets of CD4^+^ T cells [Th1, Th2, Th17, and regulatory CD4+ T cells (Tregs)]. Several miRNAs(miR-132, miR-200, miR-212a) regulates the differentiation and function of Th1 and Th2 and controls the pathology (64) (**Figure 2**). It is reported, recently, that miR-873 could facilitate the differentiation of CD4^+^ T cells into Th17 lineage by targeting Foxo1. miR-873 expression was significantly up-regulated in patients with SLE, and its expression was positively associated with disease severity (65). miR-124 suppresses the immune activity of CD4 + T cells from SLE patients by downregulating interferon regulatory factor 1(IRF1) (66). miR-301a-3p was found significantly upregulated in peripheral blood mononuclear cells (PBMC) from SLE patients, and the increasing miR-301a-3p promoted IFN-gamma, IL-17, and IL-6 expression in PBMC *in vivo* (67). miR-4512 expression was significantly decreased in PBMC and macrophages of SLE patients. miR-4512 regulates the level and type of chemokines by TLR4 and C-X-C motif chemokine ligand (CXCL)2 pathway, which point out that the miR-4512-TLR4-CXCL2 axis as a potential novel therapeutic target in SLE (68). miR-let-7b is a TLR-7 agonist and plays a role in the induction of proinflammatory responses in vascular cells by proinflammatory neutrophil (low-density granulocyte (LDG)) nets in SLE (69). Downregulation of miR-125a attenuated the suppressive effect of IL-16 gene, thereby up-regulating neutrophil chemokine expression in lung epithelial cells, resulting in lung injury in SLE (70).

Follicular T helper (Tfh) cells play a great role in mediating the interaction between T and B cells with their important surface molecules for mediating the interaction between T and B cells. miR-17-92 cluster has a negative and positive effect on Tfh cell differentiation. Additional studies found that regulation of Tfh cell differentiation by miRNA makes difference in maintaining immune tolerance and preventing SLE (71). Geng, Tang et al. reported recently that miR-663 impairs BMSC-mediated downregulation of Tfh cells and upregulation of Tregs by targeting transforming growth factor β1 (TGF-β1) (72). And miR-663 is considered as one of the candidates of targets for therapies.

Direct damages caused by autoantibodies and immune complexes in the inflamed tissues are the characteristic features in SLE. Association between miR-30a, miR-155, miR-181b, miR-15a, and hyperactive B cells suggests that miRNAs are the major regulator in the production of autoantibodies (73). Luo, Ding et al. found that Kruppel-like factor 5 (KLF5) was a direct target of miR-152-3p, and it could bind to the promoter region of BAFF and inhibit its expression in B-cells. It means miR-152-3p participates in the disease process of SLE by regulating B-cell self-reactivity and autoantibody production (74).

### Exosome-derived miRNA and its function in SLE

Valadi et al.found that mRNA and miRNA were contained in exosomes for the first time (75). There is generated immense evidence showing that exosome-derived miRNA has tightly relevance to the immune system and autoimmune diseases.

Exosome is a small lipid vesicle that can contain protein and nucleic acid, which can be released by different immune cells, including B cells, T cells, DC cells, and mast cells (76). Exosome is just one particular type of extracellular vesicles (EV), and they are divided according to the diameter, which technically include exosome(30-120nm), microvesicles(100-1000 nm), and apoptotic bodies (50-5000 nm). Apart from different size and characteristics in morphology, these three types of extracellular vesicles also vary in biochemical composition and biogenesis (77, 78). One of the exosomes’ functions may be specific interaction with the target recipient cell, enhancing the communication among different cells by delivering cargo, and strengthening the spread of immune factors.

Different origin exosomes can facilitate the immune response (79), it’s the function in antigen presentation, inflammation, programmed cell death, and angiogenesis, has been carefully studied (80). An increasing number of studies have proved that miRNA transferred by exosomes to recipient cells can precisely affect the target genes. Subsequently, the specific miRNA of exosome triggers functional responses in the target cells such as lymphocytes, monocytes, and neutrophils. Meanwhile, these activated forms of target cells with increased IFNR1 (Interferon Alpha And Beta Receptor Subunit 1) expression may exert beneficial roles in regulating the function of the adaptive and innate immune systems (81–87). Mauro Poggio et al. found that exosome can suppress the immune response, and its genetic blockage enhances T cell activity in the draining lymph node to induce systemic immunity and memory (88). Through the epigenetic modifications, miR-126, miR-148a, and miR-21 derived from SLE patients’ exosome can alter the autoimmune-associated genes’ expression (89).

The majority of urinary miRNAs are contained primarily in exosomes in SLE, including miR335-5p, miR-302, miR-200c, and miR-146a. Among these exosomal miRNAs, only miR-146a was found to discriminate active LN (90). miR-146a has an association with local inflammation, and miR-26a is derived from urine exosomes involved in renal injury in LN (91).Laura Claßen et al. reported that the levels of miRNA (miR-155*, miR-34b, and miR-34a) derived from microvesicles in T lymphocytes were deregulated in SLE when compared to healthy individuals (92). This dysregulation of the expression of distinct miRNAs may be associated with the development of SLE. Valentina Salvi et al. found that exosomes derived from the plasma of SLE patients can increase the secretion of IFN-α by human blood pDCs *in vitro*. Further investigation clarified that miRNA isolated from exosome can work as self-ligands of innate single-stranded endosomal RNA sensors, providing the potential capacity of exosome-derived miRNAs as novel TLR7 endogenous ligands to induce pDC activation as well as potential therapeutic targets in SLE (93).

Accumulating evidence showed that exosome have been found in many body fluids, including blood, urine, breast milk, and saliva (76). Due to their specific protein, RNA, and lipid containing and competitive non-invasive diagnostics methods, exosomes have attractive potential to be applied in clinical. Exosome-derived miRNAs can be used as biomarkers to provide an innovative therapeutic approach due to their non-invasive and accurate detection. The down-regulated serum expression level of exosomal miR-451a was negatively correlated with SLEDAI score and kidney damage (94). A single-center study found that exosomal miR-146a expression was significantly downregulated and negatively correlated with anti-dsDNA antibody levels and ESR in SLE patients; Conversely, miR-21 and miR-155 were significantly elevated and positively correlated with proteinuria, which indicated that they may serve as potential biomarkers for the development of LN (95). The level of miR-183-5p expression in the PBMCs of SLE patients was positively correlated with SLEDAI-2000 and the amount of anti-dsDNA antibody by negatively regulating transcription factor (Foxo1) expression, which can be used as SLE biomarkers (96). Also, several miRNAs (miR-485-5p, miR-132, miR-145, and miR-183) have been suggested as promising SLE biomarkers (97–99). Exosomes have the advantage of low immunogenicity, and various routes of administration, and high stability in blood as drug carriers for drug delivery. It can act as a gene therapy vector or carry therapeutic RNA to target cells (100).

Javier Perez-Hernandez reported that miR-146a derived from SLE patients’ urine exosomes can discriminate the presence of active lupus nephritis (90). Many researches are constructing a drug delivery system to send specific content using exosomes. Alvarez-Erviti L et al. experimented brain-delivery of specific miRNAs on the mouse model (101). During the past decade, some methods of EV or exosome have been applied in the therapeutic of treating autoimmune and inflammatory diseases, such as EVs derived from mesenchymal stem cells and antigen-presenting cells (102).

### Effects of miRNAs on DNA methylation in SLE patients

**Figure 3:**
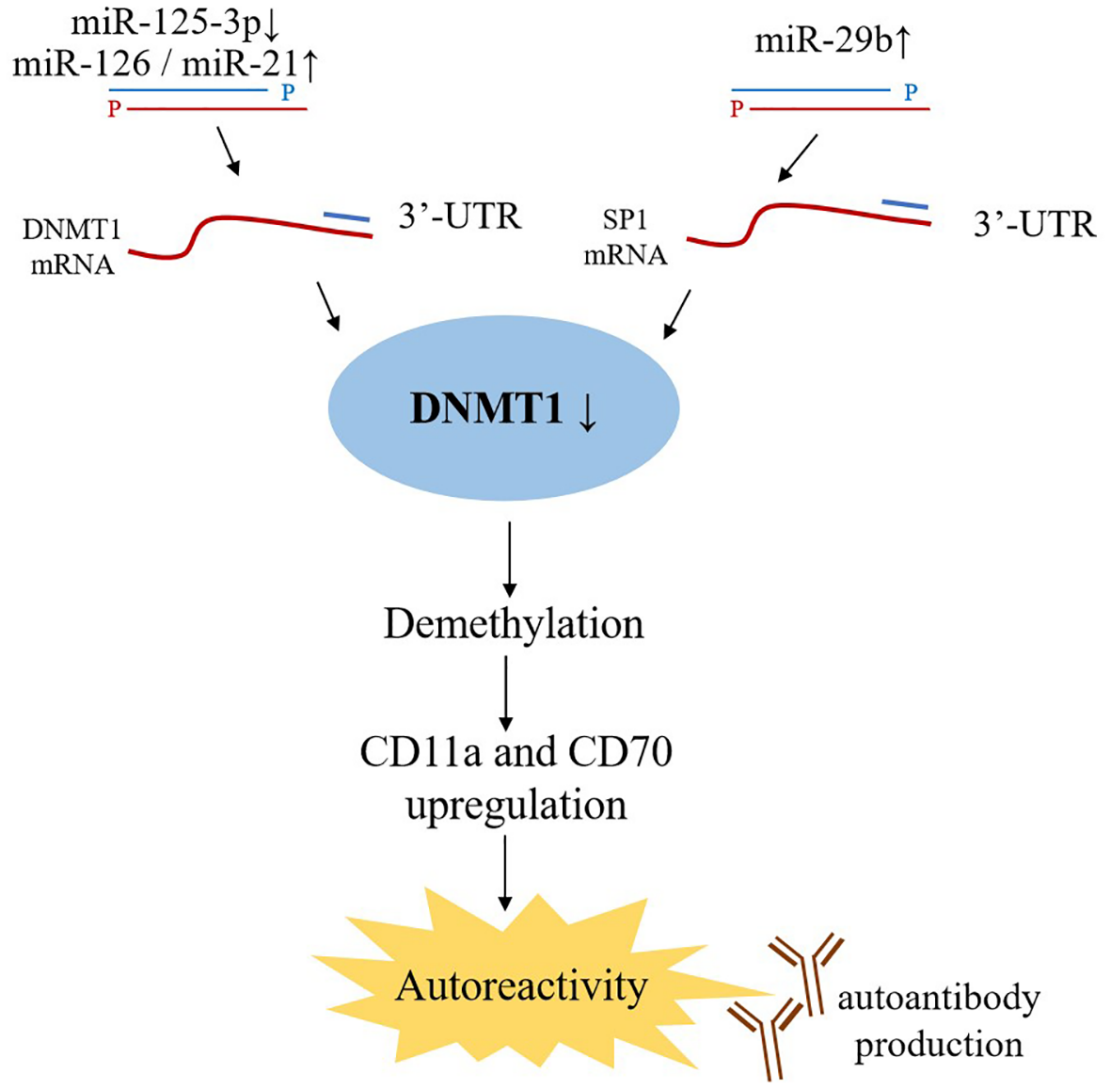
Effects of miRNAs on DNA methylation. miR-21 and miR-126 binding to the 3’ UTR in DNMT1 mRNA lead to DNMT1 protein decreased, while miR-29b targets 3’ UTR of SP1 mRNA indirectly inhibiting DNMT1 expression. As a key component of DNA methylation, decreased DNMT1 expression upregulates the level of CD11a and CD70, which ultimately trigger autoreactivity and amount of autoantibodies production.

The main epigenetic processes include DNA methylation, post-translational histone modifications, and noncoding RNA (miRNAs, lncRNA, and siRNA) that regulate gene expression. Recently, epigenetics’ role in the field of SLE has called for tons of dedication. It has been reported that several miRNAs, especially miR-21 and miR-126, can control the transcription of DNMT1 (DNA methyltransferase 1), a key component of DNA methylation (**Figure 3**). Among them, miR-21 can target DNMT1 as well as inhibit the RAS-MAPK-ERK signaling pathway upstream of DNMT1 in T cells (103). In CD4^+^ T cells of SLE patients, miR-126 and the sponge(hsa_circ_0012919) for miR-125-3p were found abnormal expression, which results in a reduction of DNMT1 by targeting 3′- UTR of DNMT. Decreased DNMT1 caused the demethylation and up-regulation of methylation-sensitive genes encoding CD11a and CD70, which are proportional to disease activity (89, 104, 105).

miR-29b, also upregulated in SLE CD4+ T-cells, can negatively regulate DNMT1 expression by targeting SP1; further studies demonstrated that inhibition of miR-29a in the T-cells of SLE patients reversed DNA hypomethylation and the upregulation of downstream genes (34). These findings provides potential novel strategies for therapeutic interventions.

### Considerations for miRNAs as therapeutic targets in SLE

Both specific up-regulation and down-regulation miRNAs are potential therapeutic targets

in SLE (106–108). Targeted vector design is the key to achieving the clinical application of miRNAs in the treatment of SLE, but the cross-talk between cell signaling pathways needs to be considered when developing effective therapeutic strategies. How to increase local drug concentration in carriers, improve efficacy, and reduce side effects is a test. On the other hand, miRNAs can regulate gene networks involving multiple signaling pathways, therefore, the possible additional immunostimulatory effects, off-target effects, nonspecific inflammatory effects, etc. need to be considered when applying miRNAs therapy in clinical practice (109). Recently, researchers found that SLE can be modulated by epigenetics through DNA methylation, posttranslational histone modifications, and noncoding RNA (110). Epigenetic processes are tightly associated with miRNA biogenesis and SLE pathogenesis, which is an innovative insight into therapies for SLE.

Existing research results show that miRNA can be used as biomarkers for the diagnosis of SLE and help to assess the progression of the disease extent and prognosis. At the same time, because of its unique advantages such as low immunogenicity and transmembrane ability, miRNA has broad application prospects as therapeutic drugs. At present, there are relatively few studies on miRNA as a drug in the treatment of autoimmune diseases, including SLE. Therefore, future studies aim to reveal the exact mechanism of miRNA action and the involved signaling pathways in autoimmune diseases, and further expand the studies *in vitro* or animal models to ensure the safety and effectiveness of miRNA in the diagnosis and treatment of SLE.

## Conclusion

The procedure of miRNA generation and its function can be regulated by many factors. Based on the miRNA biogenesis process, RNase-III-type enzyme, Drosha, Dicer, AGO2, RISC and other factors can affect the miRNA biogenesis, transportation and maturation. During these procedures, many other factors are vital to modulate miRNA processing and expression, such as transcriptional and post-transcriptional regulation, and epigenetic control (DNA methylation, DNA hydroxymethylation). SLE is a multiple system and organism involvement disease with complex etiology and clinical manifestation. Both innate and adaptive immune systems participate in the pathogenesis of SLE. In the past few years, many researchers have proved that miRNA can modulate the immune system by targeting pro-inflammatory cytokine production, immune cells, IFN signal pathway, etc. Recent studies claimed that exosome-derived miRNA can also regulate the immune system and have a close relationship with SLE. Due to its specific content and the superiority of non-invasive diagnostics, exosome-derived miRNAs have a potential to be the biomarker and target of SLE.

The identification of precise mechanisms and regulation of miRNA biogenesis and its interaction with the immune system and SLE pathogenesis accelerate the translational application in clinical and provoke great passion about investigating treatments for SLE. Nevertheless, translational usage of miRNAs related targets in clinical trials is still mystic and requires unprecedented dedication from laboratory to clinical investigation. Yet further advantages have been gained rapidly at present, prudently, and hopefully, we anticipate more groundbreaking progress in this area and advance together towards new tools for treating SLE.

## Author contributions

Conception and design: BL, HZ and ZZ. Data curation: all authors. Formal analysis: HZ, KZ, YL and XH. Investigation: all authors. Methodology: all authors. Project administration: BL, KZ and ZZ. Resources: all authors. Data analysis: YL, XH and KZ. Supervision: BL, HZ, YL and KZ. Writing—original draft: BL, KZ and ZZ. Writing—review and editing: BL, ZZ and XH. All authors contributed to the article and approved the submitted version.

## Funding

This work was financially supported in part by research grants from the Sanming Project of Medicine in Shenzhen(SZSM201602087), Shenzhen Science and Technology Project (JCYJ20180302145033769), The research on public welfare project in Futian District, Shenzhen (FTWS 2021062).

## Conflict of interest

The authors declare that the research was conducted in the absence of any commercial or financial relationships that could be construed as a potential conflict of interest.

## Publisher’s note

All claims expressed in this article are solely those of the authors and do not necessarily represent those of their affiliated organizations, or those of the publisher, the editors and the reviewers. Any product that may be evaluated in this article, or claim that may be made by its manufacturer, is not guaranteed or endorsed by the publisher.
